# Nosocomial pneumonia in the postoperative period after heart transplantation

**DOI:** 10.1186/cc11983

**Published:** 2013-03-19

**Authors:** R Gómez López, P Fernández Ugidos, P Vidal Cortes, M Bouza Vieiro, J Miñiz, S Fojon Polanco, M Paniagua Martin, R Marzoa Rivas, E Barge Caballero, M Crespo Leiro

**Affiliations:** 1 Complexo Hospitalario Universitario de Ourense, Spain; 2 Complexo Hospitalaro Universitario de A Coruña, Spain

## Introduction

Infections are a major complication during the postoperative period after heart transplantation (HT). In our hospital, nosocomial pneumonia is the most frequent infection in this period. The objective of this study is to determine the epidemiological and microbiological characteristics of this disease in our centre.

## Methods

A descriptive retrospective study of all medical records of HT performed in a single institution from 1991 to 2009 followed until June 2010. Clinical and microbiological variables were considered. Centre for Diseases Control (CDC) criteria were used to define nosocomial infections. Invasive aspergillosis was considered if there were criteria for probable aspergillosis according to IDSA criteria.

## Results

In 594 HTs there were 97 infectious episodes in 75 patients (12.6%). Eighty-five patients (14.3%) died during hospitalization. Infection is the second cause of mortality during the postoperative period (17.9% of dead patients). The most common locations of infections were pneumonia (*n *= 31, 31.9% of infection episodes), bloodstream (*n *= 24, 24.7%), urinary tract (*n *= 14, 14.4%), surgical site (*n *= 13, 13.4%) and intraabdominal infections (*n *= 13, 13.4%). Patients with pneumonia were treated according to knowledge in a specific moment, thus different antibiotics were used. The duration of antibiotic therapy was 20 ± 15.5 days. In nine episodes of pneumonia according to the CDC no germ was isolated in the cultures. Six of the episodes were polymicrobial infections. The most frequent microbes isolated were *E. coli *(*n *= 7, 22.5% of pneumonia cases), *A. fumigatus *(*n *= 7, 22.5%), *S. aureus *(*n *= 3, 9.68%), *P. aeruginosa *(*n *= 3, 9.68%), *P. mirabilis*, *K. pneumoniae*, *E. cloacae*, *E. faecalis*, *C. glabrata*, and *S. marcescens *(one case each, 3.22%). Pneumonia was suspected but not confirmed in 75 patients. Despite this, antibiotic treatment was maintained for a media of 17.353 ±3 7.01 days: 56 wide-spectrum treatments and 18 targeted therapy after knowing the antibiogram. The length of ICU stay was 38.4 ± 70.8 (3 to 264) days, of hospital stay was 66.2 ± 80.5 (3 to 304) days and of mechanical ventilation was 27.3 ± 50.2 (3 to 264) days. The mortality of patients with pneumonia was 32.3%.

## Conclusion

Nosocomial pneumonia is the most frequent infection in our series. Despite when infection was not confirmed, antibiotic therapy was maintained in suspect cases. We found a high incidence of aspergillosis. Limitations because of wide duration of this study should be considered.

